# Development of the Platform for Three-Dimensional Simulation of Additive Layer Manufacturing Processes Characterized by Changes in State of Matter: Melting-Solidification

**DOI:** 10.3390/ma15031030

**Published:** 2022-01-28

**Authors:** Dmytro S. Svyetlichnyy

**Affiliations:** Faculty of Metals Engineering and Industrial Computer Science, AGH University of Science and Technology, al. Mickiewicza 30, 30-071 Krakow, Poland; svetlich@metal.agh.edu.pl

**Keywords:** Additive Manufacturing, Selective Laser Melting, Lattice Boltzmann Method, Cellular Automata, modelling, solid-liquid phase transition

## Abstract

A new platform for three-dimensional simulation of Additive Layer Manufacturing (ALM) processes is presented in the paper. The platform is based on homogeneous methods—the Lattice Boltzmann Method (LBM) with elements of Cellular Automata (CA). The platform represents a new computer-based engineering technique primarily focused on Selective Laser Melting (SLM) technology. Innovative computational strategies and numerical algorithms for simulation and analysis of entire powder bed-based technology with changes in state of matter (melting-solidification) are presented in the paper. The models deal mainly with heat transfer, melting and solidification, and free-surface flow. Linking LBM and CA into a complex holistic model allows for complete full-scale simulations avoiding complicated interfaces. The approach is generic and can be applied to different multi-material powder bed-based SLM processes. A methodology for the adaptation of the model to the real material (Ti-6Al-4V alloy) and processing parameters is presented. The paper presents the first quantitative results obtained on the platform and shows the ability of the model to simulate and analyze a very complex technology, entirely without a complicated interface between the sub-models. It solves the large-scale problem connected with computer-aided design and analysis of new multi-passes and multi-materials processes.

## 1. Introduction

Additive Manufacturing (AM) is a rapidly developing area of new technologies. A review can be found, for example, in [1]. The modeling of such technologies is a novel challenge. The model is needed for the control, optimization, research, modification, invention, and development of technologies.

Many models consider only one or several phenomena of AM technologies. For example, Yan et al. [2] developed a model based on FEM for thermal multiphase flows. The combination of the level-set method and residual-based variational multiscale formulation is used. Melting and solidification are considered, but the model cannot consider the entire AM process or its cycle.

The SLM process modeled by a Level Set Finite Element method at the scale of bead formation is presented by Chen et al. [3]. A volume heat source is considered as the local absorption of the laser-transparent material with a continuous powder bed. A volume shrinkage is modeled by a compressible Newtonian law. The process was examined with a relatively large laser spot and intermediate laser power. The powder properties were homogenized.

A multi-physics simulation model to simulate a wide range of laser processes is presented by Otto et al. in [4]. This is the Finite Volume approach to solving systems of coupled partial differential equations. Results showed the feasibility of combining thermo-mechanics with a Volume of Fluid (VoF) approach. They did not consider approaches related to powdered materials.

Macroscopic thermal modeling of the Selective Laser Melting process on the scale of the constructed workpiece using the 3D finite element method is presented by Zhang et al. in [5]. They applied the model to a process with a relatively large laser spot, intermediate laser power, and low velocity. This allows homogenization of the powder properties, but it cannot be extended to other cases. They also noted very high computation times.

Thijs et al. [6] studied an evolution of the microstructure of the Ti–6Al–4V alloy processed by SLM and the influence of the scanning parameters and the scanning strategy on the microstructure studied.

Gu and He [7] presented a three-dimensional transient FEM model to predict the stress distribution of parts shaped by SLM technology. Because the objective was the study of residual stresses, the SLM model was rather simplified.

Yu and Zhao [8] presented a semi-coupled resolved Computational Fluid Dynamics (CFD) and Discrete Element Method (DEM) to simulate thermally induced phase changes and particle–fluid interactions. They employed an immersed boundary (IB) method for fluid flow modeling. The application of different simulation methods led to a relatively complicated interface that resulted in high computational efforts.

Lee and Zhang [9] modeled heat transfer, fluid flow, and solidification in processes such as SLM–laser powder bed fusion that closely resembles the weld metal microstructure but at a much finer scale. They combined a powder packing model based on the DEM and a 3-D transient heat and fluid flow simulation. The experimental data on the size of the molten pool and the solidification microstructure are compared with the corresponding simulation results.

A transient three-dimensional beam-matter interaction model was developed to simulate the process of laser beam melting of metals in the powder bed by Gürtler et al. [10]. For powder bed generation, an external code generating a regular configuration with a face-centered cubic system of spherical particles was used. Such an artificial powder bed limits realistic simulation.

An algorithm to use the Lattice Boltzmann Method to solve free surface thermal flow problems with changes in solid/liquid phase was developed by Attar and Körner [11]. A multi-distribution function model is applied to simulate hydrodynamic flow and the coupled thermal diffusion–convection problem. The model can be applied to problems with non-isothermal motion and simultaneous solidification of fluids with a free surface, including SLM.

A model for grain structure simulation during Additive Manufacturing can be based on Cellular Automaton and the Lattice Boltzmann Method, e.g., [12]. In this paper, a 2D model was developed to simulate grain structure evolution during powder-bed-based, layer-by-layer Additive Manufacturing.

Zhang et al. in [13] modeled the laser powder bed fusion (LPBF) of TiAl-based alloys. The powder as a continuous medium with homogeneous properties was considered.

The 3D Smoothed Particle Hydrodynamics (SPH) model presented by Dao and Lou [14] can be applied for simulation of Laser-Assisted Additive Manufacturing (LAAM) processes, including SLM. The model can consider the transient powder–laser interaction, heat transfer, formation and dynamics of the melt-pool, powder–melt-pool interaction, and phase change.

Russell et al. [15] applied the SPH method to resolve thermal–mechanical–material fields in a range of Laser Fusion Additive Manufacturing processes. The authors stated that the methodology can also be used to investigate SLM processes and is a promising numerical tool for simulating laser fusion-driven Additive Manufacturing processes.

Presented above are several examples of achievements [2,3,4,5,6,7,8,9,10,11,12,13,14,15] arrived at en the route to realistic modeling of highly complicated AM technologies. The objective of the work presented in the paper is the creation of a numerical platform as a new computer-aided tool to solve the problem of modeling, analysis, and design of laser-assisted additive layer manufacturing technologies. with Selective Laser Melting as a basic technology. The new platform, principles, features, models, and results are presented in the following sections.

## 2. Platform for Modeling the SLM Process

**Figure 1:**
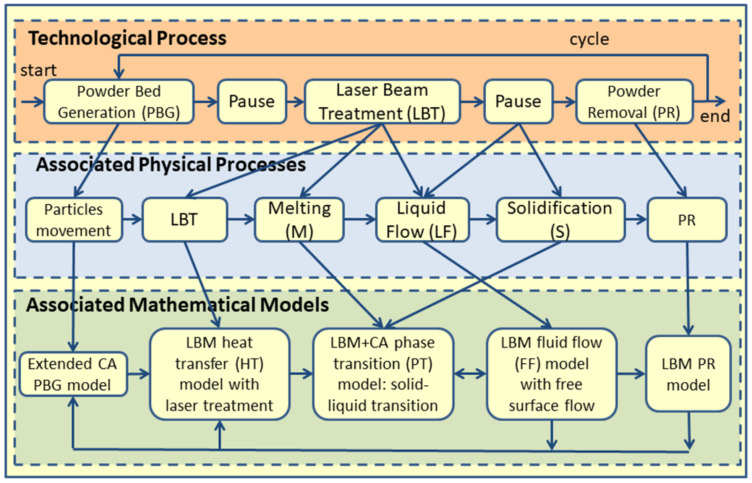
Block diagram of the SLS/SLM technological process including associated physical phenomena.

A block diagram of the technological process of SLM including associated physical processes and models presented in Figure 1.

The first conception of the modeling of some of the ALM processes presented in this paper was made by Krzyzanowski et al. [16]. They presented the idea and the first variant of the PBG model, with simulation results when filled with atomized particles.

Then Svyetlichnyy et al. [17] developed the holistic numerical model based on CA and LBM. It was devoted to laser-assisted ALM, mainly focused on SLS/SLM processes. The process was presented in four stages with a description of five phenomena and using four models (Figure 1). They included a powder bed deposition, laser energy absorption, and heating of the powder bed by the moving laser beam, leading to powder melting or sintering, fluid flow in the melted pool, flow through partly-melted or not melted material, and solidification. The main achievement was an explanation of the new conception for modeling the entire complex multi-physics process holistically and a choice of two homogeneous modeling methods that allow for the elimination of the complicated interface between different sub-models and the feasibility of realizing simulations of the whole process. There were also 1D and 2D thermal models and results for 3D gas flow.

The creation of the 2D LBM-based fluid flow model was presented in detail in [18]. This demonstrated the effect of surface tension and liquid flow on the free surface, as well as the results of melting and solidification.

The first proof of the equity of the accepted conception for a holistic model based on LBM and CA was presented in [19]. The first results of the qualitative simulation of one cycle of SLM technology was presented. The results of the 3D PBG model with atomized particles were transferred to the 2D domain, then the laser beam went through the domain and melted particles, and molten material flowed and solidified.

Paper [20] presented the further development of the numerical platform for modeling SLM. The possibilities and benefits of the proposed solution were demonstrated through a series of benchmark cases, as well as model verifications. The presented case studies dealt mainly with the melting and solidification of the powder bed, including the free surface flow, wettability with hysteresis, and surface tension. An example of process simulation showed that the approach was generic and could be applied to different multi-material SLM processes, including the solid–liquid phase transition.

The conception of the platform was extended. Variants of SLM were enhanced with those considered as multi-material processes, and examples of SLM with two materials were presented. The removal of material (particles) was added to the scheme of the SLM process, and the results of the powder removal model were presented. The modeled scheme arrived almost at the current state (Figure 1).

The last publication [21] concentrated on the thermal interaction between a laser beam and a powder bed.

In summarizing the previous achievements, it should be stressed that the numerical platform was created, the conception was formulated and proved, and qualitative simulations of the SLM cycles were fulfilled. It should be noted that all the early results presented were obtained with the use of sequential computing, and therefore in the 2D version. Further stage of the platform development would be seen as its transfer to parallel computation with the use of GPUs (graphics processing units) and implementation of the 3D version of the holistic model, as well as developing 3D versions of almost all sub-models and their verification and validation, in other words, the transition from qualitative to quantitative modeling. Some of these new achievements are presented in the following sections.

## 3. Main Algorithm

**Figure 2:**
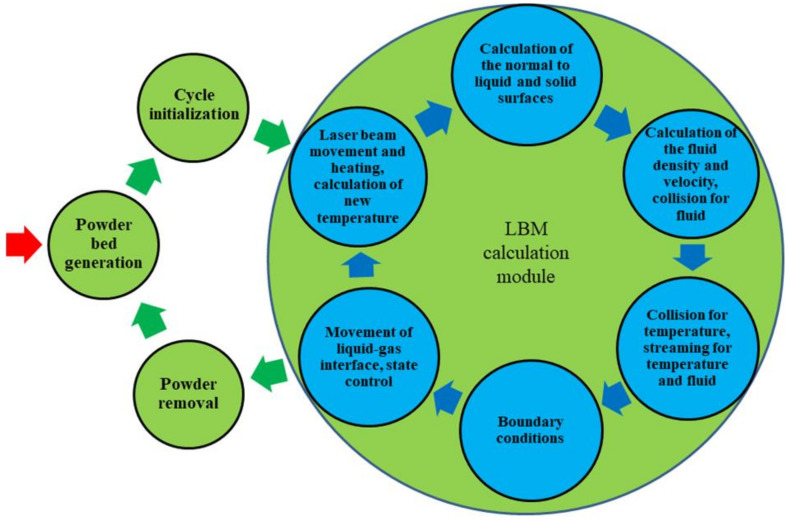
Schematic representation of the main algorithm.

The bottom part of the SLM block diagram (Figure 1) can be treated as a very common algorithm, which can be presented in more detail in the form of another block diagram of the main algorithm (Figure 2).

The new diagram has two circuits. The first represented in green is external and contains the PBG model for simulation of the powder filling, cycle initialization, and its realization (represented by the internal circuit—LBM calculation module) and the PR model (powder removal). The PBG and PR models currently communicate between themselves and with the other parts of the main algorithm with the use of files, but they should form part of a common structure with the other parts in the future. The automatic work of this circuit is still not completed. The current state of the PBG and PR models can be found in previous publications [16,21]. They are still under improvement.

The second circuit represented in blue color is internal and was realized in one pass of the SLM process. It is based on LBM with elements of CA and contains the laser beam movement, molting of the powder particles and the previously processed lower layers, flow of the molten material over and among the unmolten and semi-melted material, cooling, and solidification. The pass parameters are set in the block ‘Cycle initialization’ with the geometry prepared by the PBG model. Then the calculations in this circuit are repeated multiple times. The sub-models, subroutines, and kernels of this circuit are essential for the proper functioning of the entire platform. Some of these are described below.

## 4. Models

### 4.1. Powder Bed Generation Model

The first 3D PBG model for this platform was created and its details can be found elsewhere [16]. The model is based on 2.5D CA. The 2D CA represents a horizontal part of the 3D modeled space, while the vertical direction is independently defined in every cell. In other words, the space is discretized in the horizontal plane and continuous in the vertical direction. It operates with two kinds of particle, atomized (spherical) and of arbitrary shape. The particles of arbitrary shape are represented by a cloud of their surface points. A particle falls until it makes first contact with the basis or another particle disposed of earlier. Then it searches the second and third contact points, and stability of the found location is tested. If it is not stable, the particle moves further; otherwise, space is modified, and information is saved. The model moves to the next particle. Currently, the PBG model is being modified. Only the representation of dropping particles of arbitrary shape was changed; now they can be represented by nodes-vertices, lines-edges, and planes. The further modification will consider forces acting between particles and, perhaps, dynamics (Newton and Euler equations). However, even in its original form, the PBG shows good agreement with the experimental data [16].

### 4.2. Powder Removal Model

The PR model is still under improvement. It is created in two modifications. The first, used currently on the platform, is very simple. After the SLM cycle, all untreated particles are removed from the modeled space without considering the real process of particle removal. This seems sufficient for modeling the entire multicycles/multi-materials process. The second serves for the analysis of the real removal process but may not be very important for the platform.

### 4.3. Cycle Initialization Module

This module contains two parts: a geometric interface from the PBG model to the internal calculation circuit and an initial preparation for the internal calculation circuit.

The interface serves to adjust the geometry obtained by the CA PBG model to that of the LBM simulation module. Originally, when the simulation module was 2D, the 3D geometry was transferred as a vertical cross-section. Now, the 3D geometry is transferred as a clipping block because the PBG model can operate in a much bigger space than the LBM module; that space is highly limited by the calculation expenses. The interface can also adjust cell sizes to approach the required sizes for the particles.

The initial preparations contain calculations connected with modeled parameters: the number of iterations, the laser beam velocity, materials’ properties, and so on; they are described in the section ‘From qualitative to quantitative simulation’.

### 4.4. LBM Calculation Module

**Figure 3:**
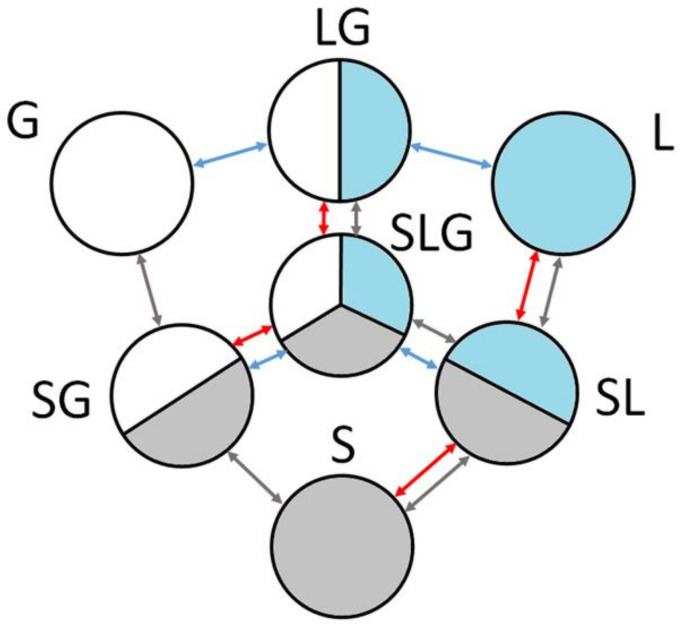
The state diagram.

First, some foundations of the LBM model should be recalled. The multistate systems can be modeled in two main ways that define the whole structure of the further applied sub-models. These approaches originate from two different methods, CA and Phase Field Method (PFM). They consider the boundaries between the materials in the different aggregate states (or states of matter) as sharp (in CA) or blurry, fuzzy (in PFM). In the PFM, the boundary is 2 to 5 cells in width and material properties are changed from one state to another continuously. In the CA, only one cell is in the boundary, and such cells are often called interfaces (e.g., liquid–gas interface), so they should only separate one subdomain from another. Properties of interface cells are changed step by step, while interface cells have properties of all neighboring subdomains. The CA boundary method is applied on the platform. This requires the use of an appropriate state diagram. This diagram developed earlier [18] is presented in Figure 3.

There are three main states of matter: Solid (S), Liquid (L), and Gas (G), and the same states of cells. There are also four types of interface cells: SL, SG, LG, and SLG, which are also defined by the fraction of matter in the solid, liquid, and/or gas state. The interface cells separate subdomains with different states of matter. Boundaries can be replaced, and this replacement is realized by the transition of the cell state. First, the fractions are changed, and when one of the fractions reaches a value below zero, the state of the cell is switched. All transitions expected in the model are shown in Figure 3 by arrows. The color of the arrows indicates an appropriate kind of transition or movement. The red arrows correspond to the thermal transition, with changes in the aggregative state of solid–liquid matter in both directions (melting–solidification). Blue arrows are related to the fluid flow with the free surface, and gray arrows to the movement of the material as a solid body. The last movement is realized only partly.

The models of the LBM calculation module serve for simulation of such transitions presented on the state diagram (Figure 3), and in such a way they allow for modeling the whole cycle of the SLM process.

### 4.5. Laser Beam Treatment Model

Currently, the old modified version of the LBT model is used on the platform. It is described in detail elsewhere [18], as well as simulation results and qualitative verification [20]. This version considers only energy transfer from the laser beam to the opaque and translucent material with varied transparency. The modification of the version presented here in comparison to the original [20] consists of the transition from the 2D to the 3D case. Now a new version of the model is under development [21]. It is expected to be more accurate because it will additionally consider such phenomena as light reflection and dispersion from the surface, and heating by reflected light, transmission with refraction and dispersion, and scattering of the light in translucent materials. However, these improvements are not crucial for modeling.

Therefore, the LBT model defines the power and location of the laser beam, the size of its spot, the intensity of radiation, and calculates the heat transfer to correspondent cells as a heat source *Q*, used further to calculate the new temperature.

### 4.6. Liquid Flow Model

The parts of the LF model enter several blocks of the algorithm presented in Figure 2—calculation of macroscopic variables (fluid density and velocity), collision and streaming operations, consideration of boundary conditions and movement of the liquid–gas interface.

The D3Q19 velocity model is applied for the flow task. Therefore 19 components of the distribution functions are used in the model that represent the following directions and velocities. C—center with velocity *v_i_* = 0; main directions with velocity *v_i_* = 1: E—east (*x*-axis, right), W—west (*x*-axis, left), N—north (*y*-axis, closer), S—south (*y*-axis, further), U—up (*z*-axis), D—down (*z*-axis); and diagonal components of three planes with velocity $v_{i}=\sqrt{2}$: NE, NW, SE, SW, UE, UW, DE, DW, UN, US, DN, DS.

The first operation of the LF model according to the algorithm is the calculation of macroscopic variables, i.e., the fluid density *ρ*, and the velocity v, according to the following:

(1) $$\rho =\sum_{i=1}^{b}f_{i},$$

(2) $$\rho v=\overset{b}{\underset{i=1}{\sum }}f_{i}e_{i},$$

where *f_i_* = *f_i_*(**x**,*t*)—distribution function of the liquid flow model, **x** = {*x,y,z*}—coordinates of the lattice nodes (or cells), *t*—time, *b*—number of vectors in the velocity model, *b* = 19, **e**—phase space variable—velocity (vector), {**e***_i_*, i = 1, …, *b*}—set of discrete velocities, velocity model, **v**—velocity vector $=\left\{v_{x},v_{y},v_{z}\right\}$, *i*—component of the velocity model.

The next operation is a collision, which calculates the output distribution function *f_i_*^out^ based on the input *f_i_*^in^ and equilibrium *f_i_*^eq^ distribution functions. The equilibrium *f_i_*^eq^ distribution function is defined as follows:

(3) $$f_{i}^{\mathrm{eq}}=w_{i}\rho [1+3e_{i}\cdot v+4.5{\left(e_{i}\cdot v\right)}^{2}-1.5v\cdot v],$$

where *w_i_*—weights for calculation of the equilibrium distribution function.

The Bhatnagar-Gross-Krook (BGK) collision operator *Ω* [22] is used here, as follows:

(4) $$f_{i}^{\mathrm{out}}=f_{i}^{\mathrm{in}}+\Omega_{i}+F_{i}=f_{i}^{\mathrm{in}}+\frac{\Delta t}{\tau }\left[f_{i}^{\mathrm{eq}}-f_{i}^{\mathrm{in}}\right]+F_{i},$$

where *F*—external force, for example, gravity, Δ*t*—time step, *τ*—relaxation time.

Finally, the streaming operation is performed according to:

(5) $$f_{i}^{\mathrm{in}}\left(x+e_{i},t+\Delta t\right)=f_{i}^{\mathrm{out}}\left(x,t\right),$$

### 4.7. Heat Transfer Model

Parts of the HT model are used in several blocks of the algorithm presented in Figure 2—calculation of macroscopic variables (temperature), collision, and streaming operations, as well as consideration of boundary conditions and phase transition in the solid–liquid interface (melting–solidification).

The same D3Q19 model is applied for the temperature task, i.e., 19 components of the distribution functions *g_i_* are used in the 3D model.

The new temperature *T* is calculated as follows:

(6) $$T=\sum_{i=1}^{b}g_{i}+Q,$$

where *g*, *g_i_*(**x**,*t*)—distribution function of the heat transfer model, *Q*—heat source.

Here, *Q* is taken from the LBT model presented above, then in the PT model the new temperature is compared with the old and the transition temperature to check whether phase transition takes place. The PH model is given below.

Then the collision operation is carried out:

(7) $$g_{i}^{\mathrm{eq}}=w_{i}T\left[1+3e_{i}\cdot v+4.5{\left(e_{i}\cdot v\right)}^{2}-1.5v\cdot v\right],$$

(8) $$g_{i}^{\mathrm{out}}=g_{i}^{\mathrm{in}}+\frac{\Delta t}{\tau }\left[g_{i}^{\mathrm{eq}}-g_{i}^{\mathrm{in}}\right],$$

The last operation is streaming; this seems to be the same as for the LF model:

(9) $$g_{i}^{\mathrm{in}}\left(x+e_{i},t+\Delta t\right)=g_{i}^{\mathrm{out}}\left(x,t\right),$$

### 4.8. Phase Transition Model (Melting–Solidification)

**Figure 4:**
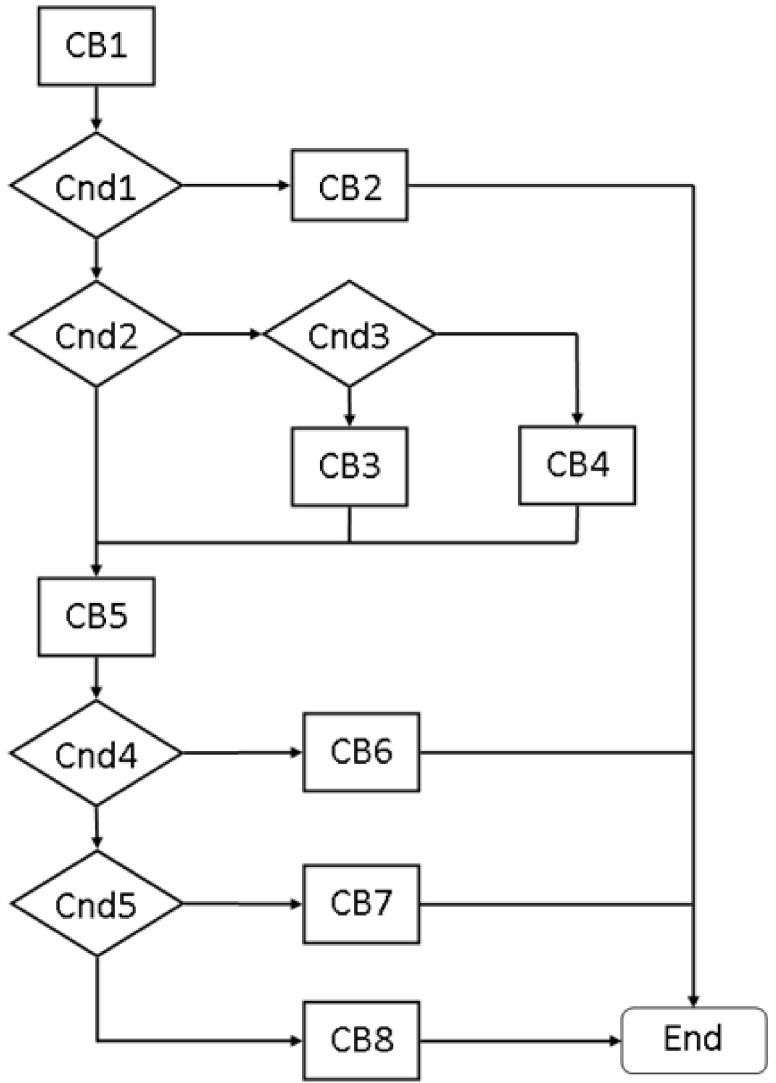
Block diagram of the algorithm of the phase transition model.

The PT model is applied to materials with non-zero specific enthalpy. An algorithm of the PT model is first shown in Figure 4.

The algorithm contains five conditional blocks, Cnd1–Cnd5, eight computing blocks, CB1–CB8, the state of the cell *q*, the previous *T*_old_ and the new *T*_new_ temperature, material data and the volume fractions of the liquid *φ_L_* and solid *φ_S_* phases at the input of the algorithm. The cell state, the new fractions *φ_L_* and *φ_S_* and the temperature *T*_new_ are seen at the output.

#### 4.8.1. Conditional Blocks

Cnd1—presence of the transition. The identification of the transition is carried out by comparing the old *T*_old_, new *T*_new_, and melting Tm temperature: (*T*_old_ − *T*_m_)(*T*_new_ − *T*_m_) > 0. Satisfying this condition leads to computing block CB2, and failing lead to Cnd2.

Cnd2—a continuation of the transition. If the old temperature *T*_old_ is equal to the melting temperature *T*_m_:*T*_old_ = *T*_m_, this is the continuation of the transition, and the algorithm goes to block CB5. If this condition is not met, the transition begins in the current iteration, moving to the conditional block Cnd3.

Cnd3—beginning of the transition. This condition is used to determine whether melting or solidification begins. If the old temperature *T*_old_ is less than the melting temperature *T*_m_:*T*_old_ < *T*_m_, this represents the beginning of melting and the transition to block CB3. Otherwise, it is the beginning of solidification and goes to block CB4.

Cnd4—the end of melting. If the entire volume of the material in the cell has been melted (transferred into liquid), this is the end of melting. The volume fraction of the solid phase *φ_S_* is checked. If the fraction is less than or equal to zero *φ_S_* ≤ 0, this marks the end of the transformation, moving to block CB6. Otherwise, it goes to the conditional block Cnd5.

Cnd4—the end of solidification. If the entire volume of the material in the cell has been solidified (transferred to solid), this is the end of solidification. The volume fraction of the liquid phase *φ_L_* is checked. If the fraction is less than or equal to zero *φ_L_* ≤ 0, this marks the end of the transformation, moving to block CB7. Otherwise, it goes to block CB8.

#### 4.8.2. Calculating Blocks

CB1—calculation of the new temperature *T*_new_ from the HT model according to (6).

CB2—final temperature without transition (the fractions remain without changes):

(10) $$T\left(x,t\right)=T_{\mathrm{new}},$$

CB3—a change in cell state at the beginning of melting: S → SL or SG → SLG (see Figure 3).

CB4—a change of cell state at the beginning of solidification: L → SL or LG → SLG.

CB5—calculation of volume fractions. Calculation of the changed mass ∆*m* considering the specific latent heat *L* and the specific heat *C_p_*:

(11) $$\Delta m=\frac{C_{p}\left(T_{\mathrm{new}}-T_{m}\right)}{L},$$

Calculation of volume fractions of the liquid *φ_L_* and solid *φ_S_* phases:

(12) $$\varphi_{S}=\varphi_{S}-\frac{\Delta m}{\rho_{S}},$$

(13) $$\varphi_{L}=\varphi_{L}+\frac{\Delta m}{\rho_{L}},$$

CB6—temperature of the liquid after melting:

(14) $$T\left(x,t\right)=T_{m}-\frac{L_{\varphi_{S}\rho_{S}}}{C_{p}},$$

Correction of volume fractions:

(15) $$\varphi_{L}=\varphi_{L}+\frac{\varphi_{S}\rho_{S}}{\rho_{L}},\varphi_{S}=0,$$

CB7—temperature of the solid after solidification:

(16) $$T\left(x,t\right)=T_{m}+\frac{L_{\varphi_{L}\rho_{L}}}{C_{p}}$$

Correction of volume fractions:

(17) $$\varphi_{S}=\varphi_{S}+\frac{\varphi_{L}\rho_{L}}{\rho_{S}},\;\varphi_{L}=0,$$

CB8—temperature during transition:

(18) $$T\left(x,t\right)=T_{m}$$

### 4.9. Fluid Flow Boundary Condition and Interface

There are four boundary conditions applied in the LF model: bounce-back, symmetric, open with a constant density (pressure), and open with a constant velocity. These boundary conditions are used to reconstruct missing components of input distribution functions. The same rules are applied for the solid–liquid interfaces. For the liquid-gas interface, the curvature is additionally considered.

The bounce-back rule:

(19) $$f_{i}^{\mathrm{in}}\left(x,t\right)=f_{\bar{i}}^{\mathrm{out}}\left(x,t\right)$$

where *ī* defines the reverse direction: **e***_ī_* = −**e***_i_*. For example, for the left (east) boundary *i* = (E, NE, SE, UE, DE) → *ī* = (W, SW, NW, DW, UW).

The symmetrical boundary conditions mean that conservation of tangential velocity components (momentum) and changes of normal velocity components occur in opposite directions:

(20) $$f_{i}^{\mathrm{in}}\left(x,t\right)=f_{î}^{\mathrm{out}}\left(x,t\right)$$

where î defines a reflected direction. For example, for the left (east) boundary *i* = (E, NE, SE, UE, DE) → î = (W, NW, SW, UW, DW). This can also be a linear combination of (19) and (20).

Open boundary conditions are defined, for example, for the upper boundary by the following equation:

(21) $$\rho \left(1-v_{z}\right)=f_{C}+f_{E}+f_{W}+f_{N}+f_{S}+f_{\mathrm{NE}}+f_{\mathrm{NW}}+f_{\mathrm{SE}}+f_{\mathrm{SW}}+2\left(f_{U}+f_{\mathrm{UE}}+f_{\mathrm{UW}}+f_{\mathrm{UN}}+f_{\mathrm{US}}\right)$$

Equation (21) gives the possibility of defining the density ρ or normal velocity (here *v_z_*) if one of these variables is set or calculated in another way (e.g., by approximation).

Additionally, for the liquid–gas interface (LG and SLG), the following equation can be used:

(22) $$f_{i}^{\mathrm{out}}=f_{i}^{\mathrm{eq}}\left(\rho_{G},v_{n}\right)+f_{\bar{i}}^{\mathrm{eq}}\left(\rho_{G},v_{n}\right)-f_{\bar{i}}^{\mathrm{in}}$$

where **v***_n_* is the velocity component normal for the surface of a liquid on the boundary.

The gas density *ρ*_G_ is influenced by the gas pressure *p*_G_ and the surface curvature *κ*, which depends on the local shape of the surface and surface tension *γ*:

(23) $$\rho_{G}=3p_{G}+6\gamma \kappa$$

### 4.10. Thermal Boundary Condition and Interface

There are three boundary conditions applied in the HT model: bounce-back (insulated boundary conditions), constant temperature, and open with a temperature approximation. The interfaces consider the heat transfer coefficient *h*.

The thermal bounce-back rule seems the same as the fluid flow bounce-back rule (19):

(24) $$g_{i}^{\mathrm{in}}\left(x,t\right)=g_{\bar{i}}^{\mathrm{out}}\left(x,t\right)$$

The constant temperature *T* is introduced as follows:

(25) $$g_{i}^{\mathrm{in}}=w_{i}T-g_{\bar{i}}^{\mathrm{out}},$$

For example, for the west boundary *i* = (E, NE, SE, UE, DE) → *ī* = (W, SW, NW, DW, UW)

The thermal interface between the different materials in the different aggregative states is defined by the heat transfer coefficient *h* while an equation is a linear combination of the bounce-back rule (24) and the streaming operation (9):

(26) $$g_{i}^{\mathrm{in}}\left(x,t+\Delta t\right)=hg_{i}^{\mathrm{out}}\left(x-e_{i},t\right)+\left(1-h\right)g_{\bar{i}}^{\mathrm{out}}\left(x,t\right).$$

The first term of the right side of (26), i.e., streaming, is a heat penetration through the interface (boundary) from one material to another, the second term, i.e., bounce-back, is a heat reflection, back to the same material. The heat transfer coefficient *h* for LBM is in the range of 0 to 1. The unit of the heat transfer coefficient corresponds to the lack of thermal resistance on the boundary, while zero means the insulated boundary.

### 4.11. Mass Exchange and Movement of the Liquid–Gas Interface

The mass exchange Δ*m_i_* between the LG cell and its neighboring cells depends on the type of neighboring cell. No mass transfer is possible between an interface and stationary gas/obstacle (solid) cells:

(27) $$\Delta m_{i}=0$$.

The mass transfer between fluid and interface cells is the difference between flows inward and outward from the interface cell:

(28) $$\Delta m_{i}=f_{i}^{\mathrm{in}}-f_{i}^{\mathrm{out}}.$$

Additionally, mass transfer between two interface cells takes into account their average liquid fractions *φ_L_*:

(29) $$\Delta m_{i}=0.5\left[f_{i}^{\mathrm{in}}\left(x,t\right)-f_{i}^{\mathrm{out}}\;\left(x,t\right)\right]\left[\varphi_{L}\left(x,t\right)+\varphi_{L}\left(x+e_{i},t\right)\right].$$

After mass exchange, the new liquid fraction for the interface cells is calculated according to the expression: *φ_L_* = *m*/*ρ_L_*.

## 5. From Qualitative to Quantitative Simulation

As recalled earlier, the results presented in previous publications [16,17,18,19,20,21] were obtained for the 2D models and have a qualitative character. New 3D models are described above in previous sections. To obtain the first quantitative results, it is necessary to choose the process and material parameters, evaluate the importance of different phenomena taking place during the process, choose the length and time scale for simulation, etc.

Some parameters of the materials are collected in Table 1.

### 5.1. Length and Time Scale

The physical effect should not depend on whether we use dimensional (as in real processes) or dimensionless quantities, as in LBM simulations. The law of similarity is widely used in modeling, including LBM. When the models are not the same as the real objects, one should increase the flow velocity or decrease the viscosity in such a way that the Reynolds numbers in both systems should be the same. This means that the Reynolds number must be identical in both unit systems (the physical and lattice systems). The Reynolds number is defined as:

(30) Re=HUν,

where *H*—characteristic modeled size (height), *U*—characteristic velocity.

The kinematic viscosity *ν* is connected with the time ∆*t* (time step) and length ∆*x* (lattice size) scale according to:

(31) $$\nu =\frac{\Delta x^{2}}{\Delta t}c_{s}^{2}\left(\tau_{F}-0.5\right),$$

where sound speed in material *c*^2^_s_ = 1/3 for D3Q19 velocity model, and relaxation time in LBM simulation should be *τ*_F_ > 0.5.

All transient thermal processes depend upon a Fourier number:

(32) $$\mathrm{Fo}=\frac{\alpha t}{H^{2}}$$

The thermal diffusivity *α* is related to the simulation parameters according to the following equation:

(33) $$\alpha =\frac{\Delta x^{2}}{\Delta t}c_{s}^{2}\left(\tau_{T}-0.5\right)$$

Two Equations (31) and (33) link four unknowns: time ∆*t* scale and the length ∆x scale, and the relaxation time for the thermal *τ*_T_ and flow *τ*_F_ problems. Therefore, two of these should be set or calculated in another way.

For example, for titanium and Ti-6Al-4V (Table 1): *α*/*ν* = 2.9 and *τ*_T_ > *τ*_F_. If the relaxation time for the flow is set *τ*_F_ = 0.75, then the relaxation time for the thermal task *τ*_T_ = 1.25, and the ratio (∆*x*)^2^/∆*t* = 1.2∙10^−5^ (m^2^ s^−1^) can be calculated. The lattice size can be chosen based on the average particle size (=80 µm), ∆*x* = 4 µm. Then the time step is ∆*t* = 1.33 µs.

An analysis of characteristic flow velocity performed during the first simulation shows that the Reynolds number is much lower than the critical value for the initiation of the turbulent flow. This means that the conditions of stability of the calculation are met even for the low relaxation time (*τ* = 0.55 ÷ 0.6) in a single-relaxation time (SRT) calculation scheme (method). Otherwise, the multi-relaxation time (MRT) or cascade schemes should be applied to reduce relaxation time. This is welcome, because the lower the relaxation time, the fewer iterations are needed for simulation.

### 5.2. Thermal Convection and Buoyancy Force

For natural convection (with Bosensque approximations), the controlling parameters are Grashof (Gr) or Rayleigh numbers (Ra), The Rayleigh number defines a relation between temperature-driven buoyancy and viscous friction forces. It is calculated according to:

(34) $$\mathrm{Ra}=\frac{g\alpha_{l}\Delta TH^{3}}{\alpha \nu }$$

where *g*—acceleration by gravity, ∆*T* ≈ 1000 K—maximum temperature difference, *H* = 3.2 × 10^−4^ m. For example, for titanium or Ti-6Al-4V (see parameters in Table 1) Ra_Ti_ = 676.

The Rayleigh-Bénard convection is governed by this Rayleigh number. Because Ra < 1708, thermal differences are dissipated by fluid viscosity. Elsewise, the buoyancy overcomes dissipation, and convection occurs. Therefore, the buoyancy force for molten titanium can be omitted in the simulations, as well as for molten glass and air (nitrogen). This seems natural for the chosen microscale. Although buoyancy was introduced into the model during development, its effect is minimal.

### 5.3. Surface Tension

The Bond (Eötvös) number defines a relation between gravity and surface tension. It is calculated according to:

(35) $$\mathrm{Eo}\left(\mathrm{Bo}\right)=\frac{g\Delta \rho r^{2}}{\gamma }$$

where: *r* ≈ 20 µm—radius of droplet/bubble, ∆*ρ*—a difference of the density of the liquid and vapor phases (the density of the vapor phases can be omitted).

Because the Bond (Eötvös) number is small, gravity is negligible, and the surface tension determines the droplet shape. The shape of the droplet is spherical when it has no contact with the surface or is a spherical cap when it has contact. The small Bond and Rayleigh numbers allow the setting of higher values of these numbers in simulation by increasing the gravity force to accelerate the falling of molten material into the pool, and its property is used in the simulations.

### 5.4. Heat Transfer Coefficient

The heat transfer coefficient *h* applied in (26) can be evaluated by comparing heat transfer with thermal conductivity *λ*. Newton’s law can be presented as follows:

(36) q=hΔT

where *q*—heat flux.

Fourier’s law can be written in the form:

(37) $$q=-\lambda \frac{\partial T}{\partial x}=-\alpha \rho C_{p}\frac{\partial T}{\partial x}$$

The gradient ∂*T*/*∂x* can be approximated in the LBM as Δ*T*/Δ*x*, then the heat transfer coefficient h* in the model equals the ratio of the heat flux during heat transfer and conductivity:

(38) $$h^{*}=\frac{h\Delta x}{\alpha \rho C_{p}}=\frac{h\Delta x}{\lambda }$$

For titanium and Ti-6Al-4V it will be h* = 6.9∆*x*. It seems that this can be treated as a lack of heat transfer between material and gas. However, in the model this small coefficient is applied and, as simulations show, it was the proper choice.

### 5.5. Laser Beam Movement and Heating

The velocity coefficient *C_v_* is determined by the length and time scale:

(39) $$C_{v}=\frac{\Delta x}{\Delta t},\;\left[{\mathrm{ms}}^{-1}\right]$$

Then, the laser travel speed (for example, *v_L_* = 1 ms^−1^), corresponds to the simulated lattice speed as follows:

(40) $$v_{L}^{*}=\frac{v_{L}}{C_{v}},\;\left[-\right]$$

Take the laser power *P* (W) and the spot size *D_L_* (µm]) Then, the power density of the laser beam *p* equals:

(41) $$p=\frac{4P}{\pi D_{l}^{2}}10^{12},\;\left[{\mathrm{Wm}}^{-2}\right]$$

This means that the energy obtained by the cell in an iteration equals:

(42) $$p^{*}=p\Delta x^{2}\Delta t,\;\left[J\right],\;C_{pw}=\frac{1}{\Delta x^{2}\Delta t},\;\left[m^{-2}s^{-1}\right]\;p^{*}=\frac{p}{C_{pw}},\;\left[J\right].$$

Then the laser beam attracts the following temperature, increasing in the cell during the iteration, if the entire energy is absorbed:

(43) $$\Delta T=\frac{p^{*}}{\rho \;V_{c}\;C_{p}}=\frac{p^{*}}{\rho \;\Delta x^{3}C_{p}}=\frac{p\Delta x^{2}\Delta t}{\rho \;\Delta x^{3}C_{p}},\left[K\right].\;C_{\Delta T}=\frac{\rho \;\Delta xC_{p}}{\Delta t},\left[{\mathrm{ms}}^{-1}{\mathrm{JK}}^{-1}\right]\;\Delta T=\frac{p}{C_{\Delta T}},\left[K\right].$$

For dimensionless temperature (with melting *T*_m_ = 1 and initial *T*_0_ = 0 temperature) *C_T_* = (*T*_T_ − *T*_0_), (K) it will be:

(44) $$\Delta T=\frac{p^{*}}{\rho \;\Delta x^{3}C_{p}C_{T}}=\frac{p}{C_{\Delta T}C_{T}},\left[-\right].$$

Specific latent heat (enthalpy) *L* can be related to the increase in temperature during the heating:

(45) $$\Delta T=\frac{L}{C_{p}},\left[K\right]$$

(46) $$L^{*}=\frac{L}{C_{h}}=\frac{\Delta T}{C_{T}}=\frac{L}{C_{p}C_{T}},\left[-\right]$$

The coefficient for the heat transfer coefficient *C_α_* is defined by the length and time scale and the temperature coefficient:

(47) $$\Delta T=\frac{L}{C_{p}},\left[K\right]$$

Some possible variants of the simulated parameters for the case of Ti-6Al-4V and laser power *p* = 300 W, laser spot *D_L_* = 80 µm, and laser velocity *u_L_* = 1 ms^−1^ are collected in Table 2. Independent variables are relaxation time for fluid *τ*_F_, length scale ∆*x*, and emissivity *ε*.

The presented case is very difficult for simulation because of the very high-power density received from the laser beam. The last line of Table 2 is very important for the choice of the simulation parameters. The temperature increase should be ∆*T* < 1, and not too high above L. As can be seen, satisfied parameters can be obtained only with a low relaxation time. A similar effect can be obtained with less emissivity ε but is equivalent to a lower laser power. Another method (not presented in Table 2) of obtaining satisfactory parameters is the further reducing the length and time scale (this can be seen comparing the third and fifth columns), but this leads to significant elongation of simulation time because of the great increase in the number of elements (cells) in the same modeled space and the number of iterations (a very short time step).

The influence of low gravity can also be seen. That is why gravity in simulation is artificially increased to accelerate the falling of the molten material. This can be accepted because of the low Bond and Rayleigh numbers and the low heat transfer coefficient h* mentioned above.

## 6. Simulation Results

The simulation results of one pass of the SLM process with particles of arbitrary shape are presented in this section. Ti-6Al-4V was chosen as the material. The particles of Ti-6Al-4V are of atomized shape, but for presenting results of the PBG model, the other shape was applied. The average particle size is about 35–40 µm. The model space is *x* × *y* × *z* = 128 × 96 × 64 cells with a lattice size of 4 µm, i.e., is *x* × *y* × *z* = 512 × 384 × 256 µm. The laser travel velocity is 1 m/s. Three values of laser power are presented, 150, 200, and 300 W.

**Figure 5:**
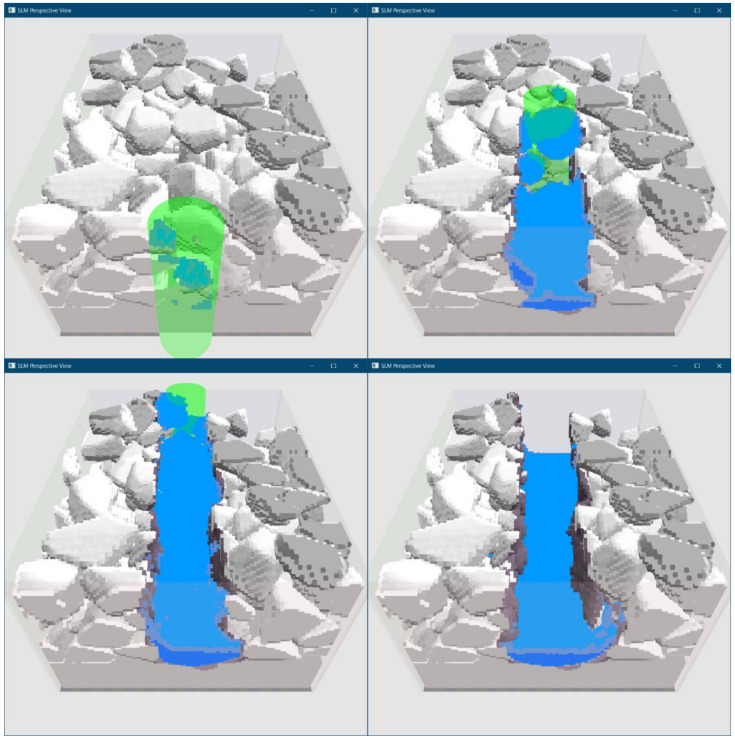
3D perspective view of the modeled process.

Figure 5 presents four stages of the pass with laser power equal to 200 W—beginning, continuation, end of the pass, and a short time after the pass. They are 3D perspective views with visible initial particle deposition presented by different shadows of gray, laser beam seen as part of the light green cylinder, and molten material shown in blue.

**Figure 6:**
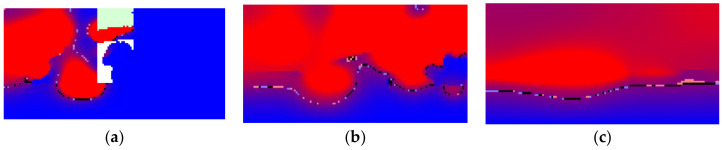
Temperature distribution in the processing—cross-section: (**a**)—at the middle of the process, (**b**)—just after the pass, (**c**)—after the droplets fall.

The temperature distribution is presented in Figure 6. The laser power is equal to 200 W. The laser beam is also shown in Figure 6a. Figure 6b presents the moment just after the pass when not all molten droplets have fallen, and Figure 6c presents the moment after the droplets fall. Red corresponds to the higher temperature (above melting temperature), and blue corresponds to the lower. Black presents phase transition temperature, and pink and light blue temperatures somewhat above and beneath phase transition temperature, respectively. The temperature distribution not at all uniform. The large difference after the pass can be explained by the fact that the laser beam energy was transferred to a different volume of the material. The temperature is higher where there were fewer particles. Here, the molten pool was also deeper.

**Figure 7:**
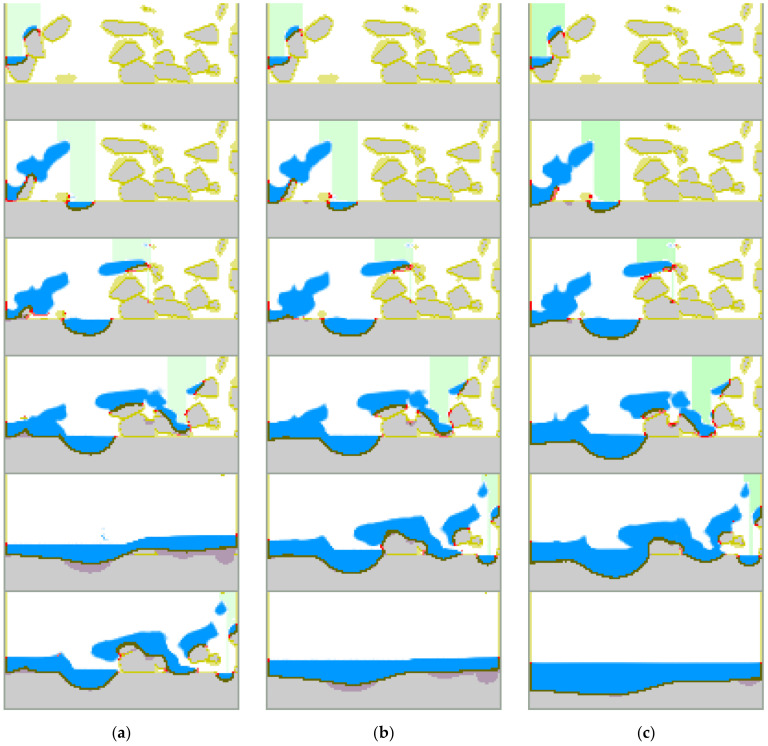
Different laser power. *p* = 150 W (**a**), *p* = 200 W (**b**), *p* = 300 W (**c**).

The state of matter is seen in Figure 7 in the same stages of the SLM pass. There are three cases presented; they differ by laser beam power. The left column presents power equal to 150, the middle to 200, and the right to 300 W. The differences between cases are not too large at the beginning of the pass, but energy is mainly accumulated not in the aggregative state but in the temperature of the liquid material, especially while the material is out of contact with the basis. Then a very hot material falling on the basis transfers its heat to the basis and melts it. The higher the laser beam power, the hotter the liquid material, and the more material of the basis is melted. At the end of the pass, the difference becomes more visible.

Analyzing Figure 6 and Figure 7, it can be seen that the atmosphere under the falling hot droplets remains cold, while above the droplets it is almost the same temperature as the melted material.

The paper presents the first 3D result obtained on the platform; calculations are fulfilled with the use of GPU and the NVIDIA CUDA environment. This allows acceleration of the calculation by several hundred times compared with CPU and the transfer of the model from 2D to 3D. For example, the calculation of one laser pass presented in the paper lasts about 20 min. This allows increasing by several times the modeled space, the resolution, or the modeling of several passes.

## 7. Discussion

The developed platform contains some sub-models, each of which required verification and validation. The previous stages of platform development consisted of separate models and their integration into a common platform [16,17,18,19,20]. The final result of these stages was a qualitative 2D simulation of one and two cycles of the SLM process [19]. The following methods are chosen among the verification methods: units tests, proof of correctness, and integration test. Model validation is based on the comparison of visual and numerical analysis, theoretical explanation, and comparison of simulation results with experimental data. Some sub-models passed all or most of the stages of verification and validation. The holistic 2D and 3D models passed the verification stages: units tests, proof of correctness, and integration test.

The simulation results presented for the first time in this paper are the last stage of verification, i.e., the integration test, and the first stage of validation of the 3D holistic model. Validation consists of obtaining the first quantitative results of modeling and qualitative comparison with similar theoretical and experimental studies.

The results obtained on this platform and partly presented in the paper were compared with the results presented by Zeng et al. in [23]. They analyzed the other 12 papers and presented their studies. A wide range of material, basement, model and sizes, laser power, scan speed, and hatch space laser spots are summarized. The finite element (FE) and finite difference (FD) methods are the most commonly used numerical methods for solving the SLM thermal problem. The main parameters which can be easily compared are the shape and sizes of the melting pool. It can be noted that the results presented here demonstrate some differences. The melting pool is somewhat narrower and longer than in most other results. This can be explained by different cooling conditions and other factors, but mainly by the difference between particle representation and properties’ homogenization. The platform presented here requires further validation, which is being prepared. This will allow for further improvement and will make the simulations more reliable and closer to the real process. Other sub-models are also expected to be improved. The next stage of model validation will be a comparison of the surface quality of the simulated and real product. For this purpose, appropriate images will be provided. However, to make a comparison, it is necessary to simulate several cycles of the filling and processing of the powder. Work in this direction is currently under way.

**Figure 8:**
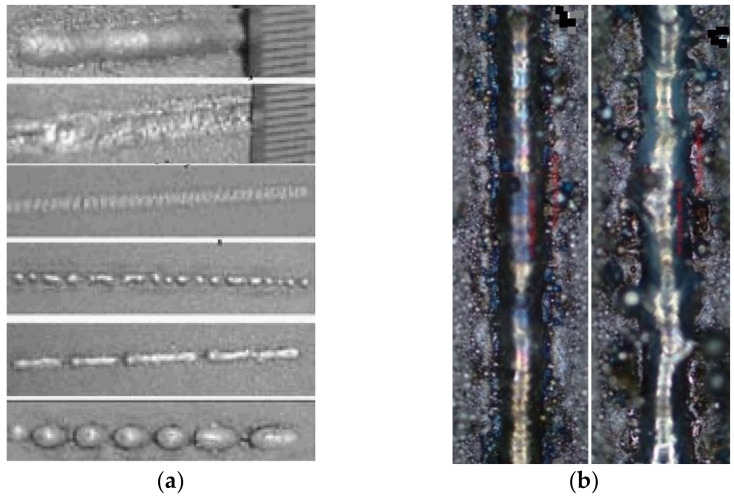
Track images: (**a**)—different laser power and scan speed [24], (**b**)—different laser power distribution [25].

**Figure 9:**
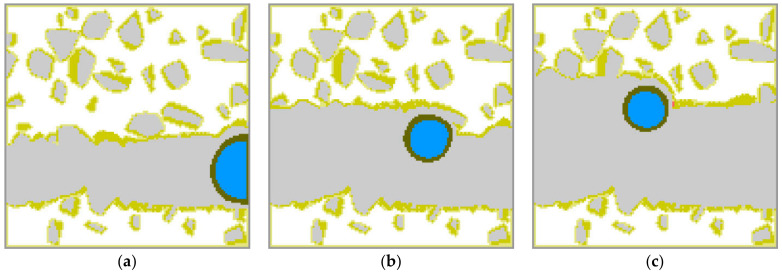
Simulated track images in three subsequent passes: (**a**,**b**,**c**)—the first, second and third passes, respectively.

Track formation depends mainly on laser beam power, scan speed, spot size, powder layer thickness, laser beam power distribution, and other process parameters. Figure 8 presents several examples of track images [24,25]. Comparing the simulated results presented in Figure 9, similarity of the track images obtained for the high power (top two images in Figure 8a,b) confirms the qualitative correctness of the model simulations.

**Figure 10:**
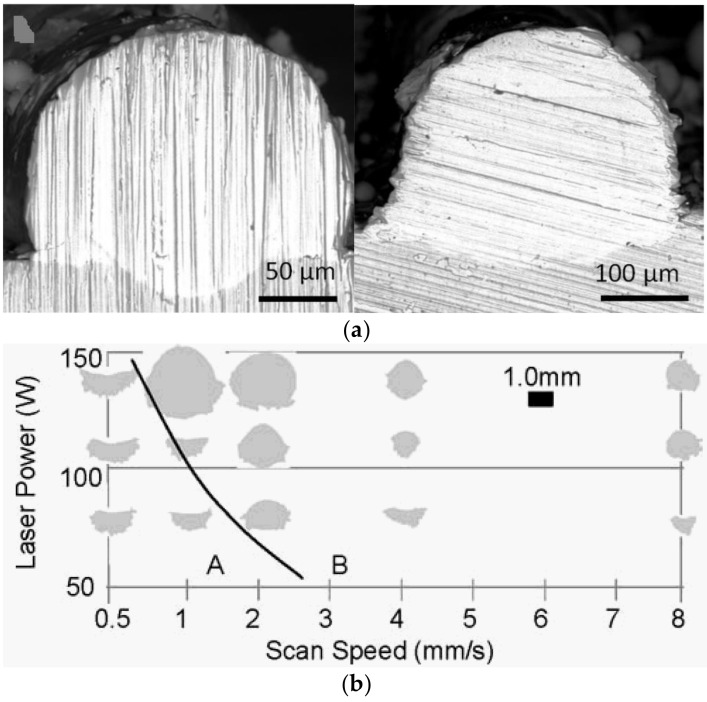
Shape of track cross-sections: (**a**)—different laser power distribution [25], (**b**)—dependence on laser power and scan speed [24].

**Figure 11:**
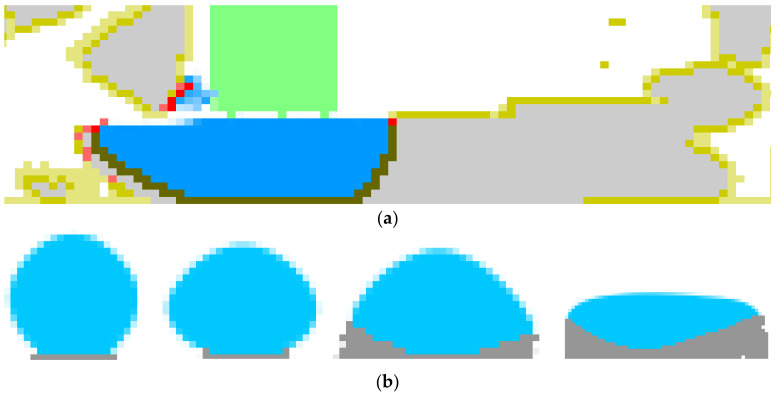
Simulated shape of track cross-section: (**a**)—during the third pass, (**b**)—different thicknesses of powder (from thick to thin).

Track cross-sections also depend on process parameters including laser beam power, scan speed, spot size, powder layer thickness, and laser beam power distribution. Examples of cross-sections are presented in Figure 10. Here SEM-analysis of the single tracks for different laser power distribution is presented (Figure 10a) [25] along with dependence of the shape of the cross-section on the laser power and scan speed (Figure 10b) [24]. Figure 11 presents shapes of the cross-section obtained in the simulations. The first image (Figure 11a) is a cross-section during the third pass when three tracks are located side-by-side (the first on the right, the third on the left), and the final surface is almost flat with a convex right side and unformed left side. Such a shape is typical of a thin powder layer and high laser power. The influence of powder thickness on the shape of cross-section obtained in simulations is presented in Figure 11b. The thicker the layer, the closer the shape will be to round. The shapes obtained in the simulations are similar to the experimental shapes of the correspondents presented in Figure 10. This confirms the accuracy of the developed model.

## 8. Summary

A new 3D platform is presented for numerical modeling of multi-material SLM processes characterized by melting and solidification of the powder material. The platform is created on two homogeneous methods: CA and LBM. That eliminates the complicated interfaces between different components of the platform and allows for modeling of the manufacturing process as well as complete full-scale calculations within the single integrated model. It differentiates the platform from the existing multiscale models based on heterogeneous numerical methods.

The platform is ready to be used for computer-aided analysis, optimization, and design of multi-material multi-pass SLM cycles.

The paper presents the following new results.

- Full description of the main sub-models which allows reproduction and repeating of the whole platform.
- The integration test of the 3D version of the holistic model.
- The first results of the 3D model, including the first quantitative results obtained on the developing platform.
- The first qualitative validation of the holistic 3D model.
- Directions for further development.

## Figures and Tables

**Table 1:** Properties of the materials at temperature 300 ÷ 1500 °C.

Properties	Ti-6Al-V	Glass	Air (Nitrogen)
Thermal expansion coefficient, *α_l_* (K^−1^)	6.5 × 10^−5^	8.5 × 10^−6^	1.22 × 10^−3^
Heat capacity, *C_p_* (J kg^−1^ K^−1^)	560	500	1 ÷ 1.25
Melting temperature, *T*_m_ (K)	1922	1336	-
Specific latent heat, *L* (J g^−1^)	360 ÷ 370	-	-
Thermal diffusivity, *α* (m^2^ s^−1^)	2.9 × 10^−6^	(37 ÷ 67) × 10^−6^	(200 ÷ 300) × 10^−6^
Convective heat transfer coefficient, *h* (Wm^−2^ K^−1^)	50	50	-
Dynamic viscosity, *µ* (Pa s)	4.42 × 10^−3^	80÷500	(30 ÷ 55) × 10^−6^
Density, *ρ* (kg m^−3^)	4420	2700	0.6 ÷ 0.35
Kinematic viscosity, *ν* (m^2^ s^−1^)	10^−6^	0.03 ÷ 0.2	(20 ÷ 250) × 10^−6^
Rayleigh number, Ra (34)	676	(0.2 ÷ 2.5) × 10^−6^	0.005 ÷ 0.1
The surface tension of the liquid-gas interface, *γ* (N m^−1^)	1.55	0.25	-
Bond (Eötvös) number, Bo(Eo) (35)	11.4 × 10^−6^	43.2 × 10^−6^	-

**Table 2:** Simulation parameters.

∆x, (µm)	4	5	5	4	4
*ε*, (-)	0.1	0.7	0.1	0.7	0.7
*τ*_F_, (-)	0.75	0.6	0.7	0.6	0.55
*τ*_T_, (-)	1.25	0.79	1.08	0.79	0.645
∆*t*, (µs)	5.33	0.0833	1.33	0.533	0.267
*L*, (-)	0.415	0.415	0.415	0.415	0.415
*C_u_*, (m s^−1^)	0.75	6	3	7.5	15
*D_L_*, (-)	10	8	8	10	10
$u_{L}^{*}$, (-)	1.333	0.166	0.333	0.133	0.0667
*g*, (-)	6.97 × 10^−6^	1.36 × 10^−6^	5.44 × 10^−6^	6.97 × 10^−7^	1.74 × 10^−7^
p*, (-)	6437	514	1030	643.7	321.8
∆*T*, (K)	3216.6	2814.5	804.1	2251.6	1125.82
∆*T*, (-)	2.05	1.794	0.512	1.435	0.718

## Data Availability

The raw/processed data required to reproduce these findings cannot be shared at this time due to technical or time limitations.
